# Novel Roles of Nestin in Postnatal Root Formation

**DOI:** 10.3390/dj13030113

**Published:** 2025-03-04

**Authors:** Yan Jing, Jinqiu Wu, Ying Liu, Xiaohua Liu, Chi Ma

**Affiliations:** 1 Department of Orthodontics, Texas A&M College of Dentistry, Dallas, TX 75246, USA; 2 Department of Biomedical Sciences, Texas A&M College of Dentistry, Dallas, TX 75246, USA; 3 Department of Chemical and Biomedical Engineering, University of Missouri, Columbia, MO 65211, USA; 4 Scottish Rite for Children, Center of Excellence in Hip, Dallas, TX 75219, USA; 5 Department of Orthopedic Surgery, UT Southwestern Medical Center, Dallas, TX 75390, USA

**Keywords:** root dentin, cementum, Nestin, odontoblast

## Abstract

**Background/Objectives:** Nestin is an intermediate filament protein and a marker of odontoblasts, but its function in tooth and bone formation is largely unknown. This study aimed to determine whether Nestin plays a role in postnatal tooth formation. **Methods:** 4-week-old Nestin knockout (KO) mice were analyzed with a range of techniques, including X-ray imaging, uCT, backscattered and acid-etched casted SEM, FITC-confocal microscopy, H&E and TRAP staining, and immunohistochemistry. **Results:** The KO mice had no apparent difference in crown formation compared to age-matched wild-type (WT) but showed delayed molar eruption with reduced TRAP^+^ osteoclasts. More importantly, KO mice developed expanded predentin and shorter, thinner roots with irregular and shortened dentin tubules. Additionally, the Nestin KO mice exhibited a reduced cellular cementum mass with sharp reductions in DMP1, OPN, and BSP. **Conclusions:** These findings suggest that Nestin plays a critical role in the postnatal development of root dentin and cellular cementum.

## 1. Introduction

The tooth is composed of the crown and root, with crown formation occurring first. This process is induced by molecular crosstalk between ameloblasts and odontoblasts, which are responsible for enamel and crown dentin formation, respectively [1]. The root formation is initiated after the crown development is largely complete. Hertwig’s epithelial root sheath (HERS), a temporary structure, plays a critical role in this process by interacting with the dental papilla cells and inducing them to differentiate into odontoblasts responsible for root dentin formation [2]. After the HERS disintegrates, dental follicle cells come into contact with root dentin and differentiate into cementoblasts, which subsequently form cellular and acellular cementum on the surface of root dentin [3].

The tooth root is crucial in anchoring teeth to the alveolar bone, while its regeneration remains a significant clinical challenge in cases of tooth trauma, agenesis, and periodontal diseases. Substantial progress has been achieved in understanding the formation and regeneration of the crown dentin [4,5]. However, the mechanisms of root dentin and cementum formation and repair remain unclear. Understanding normal root development and how it is affected under pathological conditions is fundamental for establishing efficient regenerative approaches in the oral health field.

For years, knowledge from crown dentin studies has been extensively applied to root dentin formation since both structures are formed by odontoblasts. Indeed, many signaling molecules have been found to be involved in both processes. However, significant differences in tubular density and structure, thickness, and mineralization levels between crown and root dentin clearly indicate the latter has distinct molecular specificity. For example, NFIC (nuclear factor I C) is a transcription factor essential for root formation but not required for tooth germ or crown dentin development [6,7,8]. Furthermore, recent studies, including ours, have reported alveolar bone defects in *Nfic*-KO mice, demonstrating that NFIC controls Osx expression in osteoblasts [9] and odontoblasts [10], and that Osx functions as a key downstream molecule of NFIC with an essential role in root, but not crown, dentin formation [10].

Nestin is a unique intermediate filament protein associated with the self-renewal capacity of several subsets of stem cells and progenitors [11]. Nestin expressing cells, an important subset of bone marrow mesenchymal stem cells [12], contribute to the development and regeneration of bone. Nestin is also expressed in various other tissues, including muscle [13], the cardiovascular system [14], hair follicles [15], neurons and neural stem cells [13], and kidney podocytes [16].

Recent studies on both animal models and humans suggest that Nestin is a valuable marker for odontoblast differentiation. In mice, Nestin expression was first detected in preodontoblasts in the early postnatal stage. The expression intensity gradually increased with odontoblast differentiation in both developing incisors and molars. Furthermore, Nestin was also consistently expressed in the matured odontoblasts even after the completion of dentin matrix deposition [17].

Human studies showed similar results. Nestin expression was first detected at the bell stage, where it was restricted in pulpal cells in the cusp area of fetal teeth. Nestin is found only in functional odontoblasts in young permanent teeth. However, as teeth aged, Nestin expression was progressively down-regulated and absent from older permanent teeth. Interestingly, in carious and injured teeth, Nestin expression was up-regulated in odontoblasts adjacent to the injury, indicating a potential role of Nestin in dentin repair [13].

Taken together, these studies suggest that Nestin plays a significant role in dentin formation under both normal and pathological conditions. However, few studies have explored the mechanisms by which Nestin regulates tooth development, especially root dentin and cementum formation. This study aimed to determine the role of Nestin in postnatal tooth root development by deleting the Nestin gene conventionally in mice.

## 2. Methods and Materials

### 2.1. Animals

**Figure 1:**
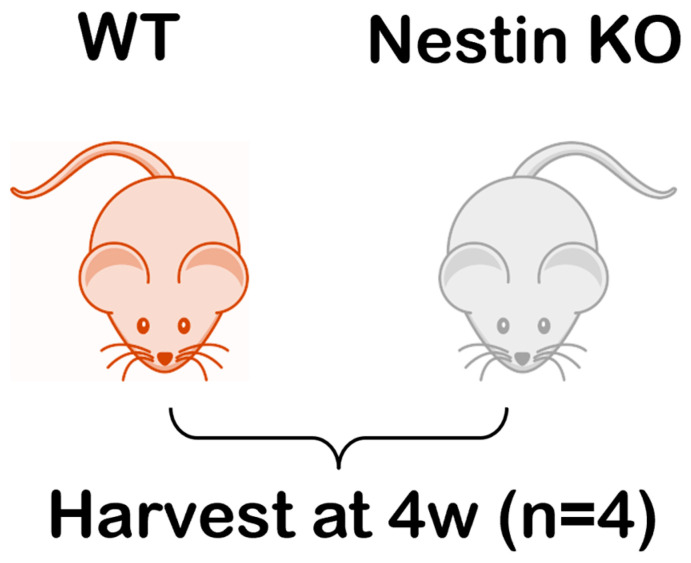
Diagram illustrating the animal model design used in this study. All mice were harvested at 4 weeks (4w).

The mandibles of 4-week-old *Nestin* knockout (KO, *Nestin* −/−) mice were generously provided by Dr. Andras Nagy [18], with wild-type (WT) mice in C57/B6 background as the controls (Figure 1). In Nestin KO mice, the *Nestin* gene was globally deleted. The number of animals per group (n = 4) was determined based on published estimates, assuming a power of 0.95 and α of 0.05, with a 10% difference and an effect size of 2.4 [10]. The experimental protocol was approved by the Institutional Animal Care and Use Committee of Texas A&M College of Dentistry (IACUC 2015-0124-CD, 30 April 2015). Animals were housed in groups within standard polycarbonate cages under 12 h day–night rhythm (7 am–7 pm) ensured by automatic dimmed lighting. Environmental conditions were maintained at a temperature of 68–79 °F and humidity of 30–70% [19]. Mice were provided ad libitum access to a standard chow diet (PicoLab Verified 75 IF Irradiated, Richmond, VA, USA) and filtered water. Enrichment materials, including nesting material and tunnels, were provided to enhance animal welfare.

### 2.2. Immunohistochemistry

The skulls of mice were fixed in 4% paraformaldehyde in phosphate-buffered saline (pH 7.4) at 4 °C for 2 days. Mandibles were carefully dissected from the skulls by removing the muscles and tendons on the surface and then separated at the midline. Samples were subsequently decalcified with 10% Ethylenediaminetetraacetic acid (pH 7.4) at 4 °C for 2–3 weeks, dehydrated in graded ethanol series (50%, 70%, 95% and 100%) and xylene, embedded in paraffin, and sectioned at 5 μm for hematoxylin and eosin (H&E) stain, Tartrate-Resistant Acid Phosphatase (TRAP) staining, and immunohistochemistry (IHC). The width measurements of predentin and dentin were obtained from an average of 4 sections per sample using Image J software (NIH, Bethesda, MD, USA).

For IHC, the following antibodies were used: anti-DMP1 and anti-BSP (Both provided by Dr. Chunlin Qin at Texas A&M College of Dentistry; 1:400), anti-Nestin (Millipore, MAB, 1:100, Burlington, VT, USA); and anti-OPN (Santa Cruz, 1:400, Dallas, TX, USA). All IHC signals were detected using a 3,3-diaminobenzidine kit (Vector Laboratories, Burlingame, CA, USA), following the instructions of the manufacturer.

### 2.3. Backscattered Scanning Electron Microscopy (SEM) and Acid-Etched SEM

The mandibles were dissected and fixed in 4% paraformaldehyde in phosphate-buffered saline (pH 7.4) at 4 °C for 2 days. Then, the specimens were dehydrated in a graded ethanol series (70%, 80%, 95%, and 100%), infiltrated with an acetone and methyl methacrylate (MMA) mixture, embedded in MMA, and sectioned along the sagittal plane through the center of the first and second mandibular molars. The exposed surface was ground with various grit sandpaper and polished with MetaDi supreme suspension of 1 μm, 0.25 μm, and 0.05 μm (Buehler, Lake Bluff, IL, USA). After 48 h of air-drying, the sample surface was coated with carbon and examined using backscattered electron imaging SEM (JSM-6010LA, JEOL, Tokyo, Japan).

For acid-etched SEM, samples underwent the same preparation steps as described above. After air-drying, the sample surface was etched with 15% phosphoric acid for 8 s, then soaked in 5% sodium hypochlorite for 20 min, repeating the process twice. Finally, samples were sputter-coated with gold and examined using secondary electron SEM (JSM-6010LA, JEOL).

### 2.4. FITC Staining

Fluorescein isothiocyanate (FITC, Sigma 46950, St. Louis, MO, USA) is a small molecular dye [20] that can penetrate dentin tubules without entering the mineral matrix. This allows to visualize dentin tubular organization using confocal microscopy. The mandibles were dissected and fixed with 4% paraformaldehyde in phosphate-buffered saline (pH 7.4) at 4 °C for 2 days. Samples were then dehydrated in a graded ethanol series (70%, 80%, 95%, and 100%), and then stained with FITC solution (1% in ethanol) for 24 h. After rinsing with 100% ethanol, the stained specimens were infiltrated with an acetone and MMA mixture and embedded in MMA. The sample blocks were sectioned along with the sagittal plane to expose the center of the first molar. A 300–400 μm thick cross section was cut, sanded, and ground to a final thickness of 30–50 μm for confocal imaging (SP5, Leica, Wetzlar, Germany) at a wavelength of 488 nm (green).

### 2.5. Statistical Analysis

The statistical analysis was conducted using PRISM. Between-group comparisons were made using independent *t*-tests to detect significant differences. A post hoc analysis was conducted to evaluate the sufficiency of the sample size.

## 3. Results

### 3.1. Nestin KO Mice Displayed Short and Thin Molar Dentin with Delayed Molar Eruption

**Figure 2:**
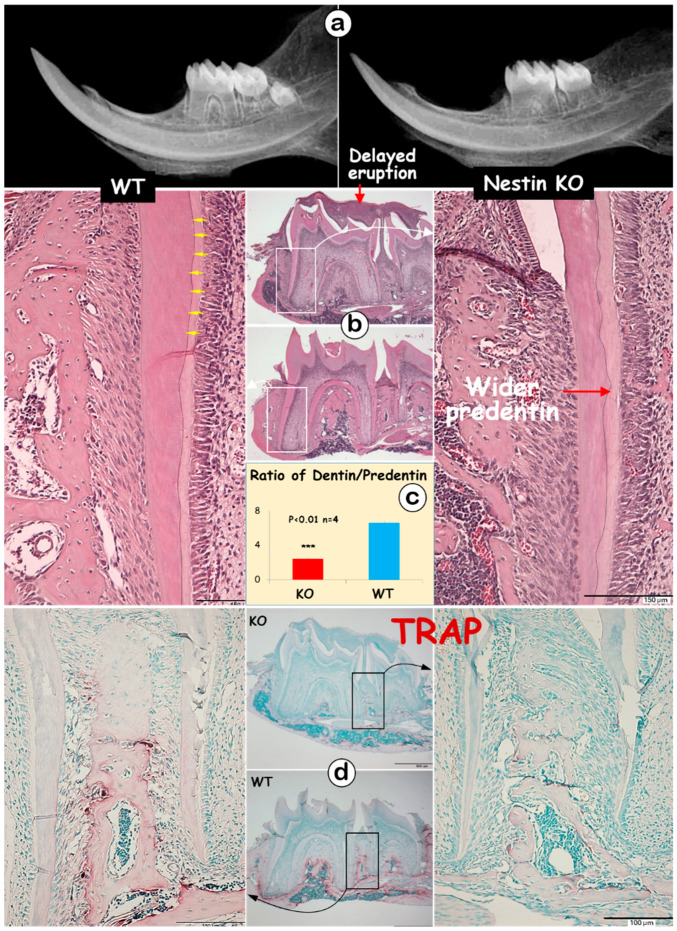
Nestin KO mice exhibit short and thin molar dentin with delayed molar eruption. (**a**) The X-ray images of 4-week-old KO and WT mice showed shorter tooth length and enlarged root pulp cavity in KO mice; (**b**) H&E staining displayed thinner dentin lacking dentin tubules (yellow arrows), wider predentin, and excessive matrix at the tops of KO molars; (**c**) the quantitative data indicated a significant decrease in the dentin-to-predentin ratio in KO mice; (**d**) TRAP staining showed fewer TRAP^+^ cells in the bone of KO mice.

The radiographic images displayed thin and short roots with expanded pulp in KO molars (Figure 2a). The H&E images showed no apparent change in the crown dentin thickness; however, KO mice exhibited a thinner root dentin with a greatly expanded predentin layer (Figure 2b). This expansion resulted in a significant decrease in the dentin-to-predentin ratio in KO roots (Figure 2c, 2.45 ± 0.08 in KO vs. 6.59 ± 0.20 in WT). Additionally, dentin tubules were clearly visible from the H&E image of the WT mice (Figure 2b, yellow arrows), but absent in KO dentin. Large areas of the matrix were observed at the tops of the 1st and 2nd KO molars, indicating a failure in removing bone matrices surrounding the molars (Figure 2b). The TRAP images, which reflect osteoclast activity, revealed fewer TRAP-positive cells in the KO mandible, which may partly explain the observed delay in tooth eruption (Figure 2d).

### 3.2. Nestin KO Mice Displayed Malformed Dentin Tubules in Roots

**Figure 3:**
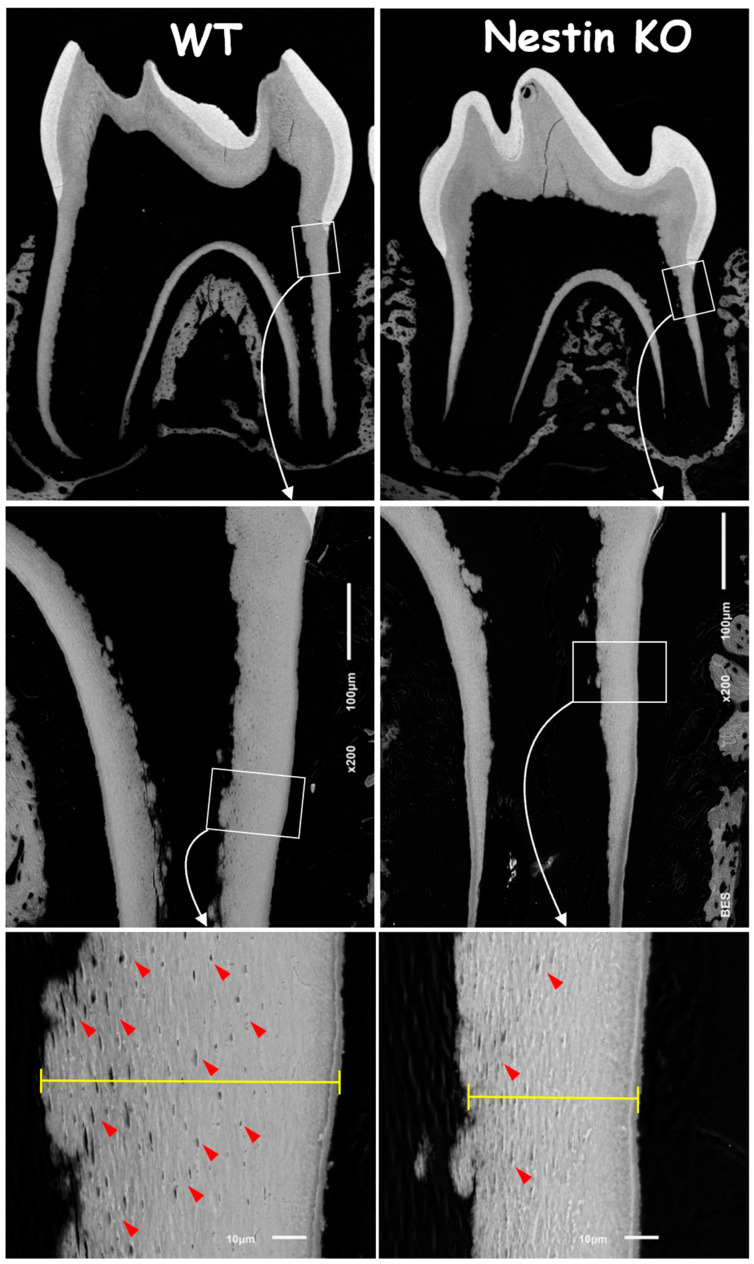
Backscattered SEM images showed a significant reduction in root dentin thickness and fewer tubular holes (red arrows) in Nestin KO mice, while no apparent differences were observed in crown dentin between groups.

To address the causative factors in root dentin defects, we first analyzed backscattered SEM images. These images showed no apparent change in the crown dentin of KO mice but revealed an obvious reduction in both the dentin width and number of tubules, as indicated by the “mini-holes” in the root dentin wall (Figure 3, red arrows).

**Figure 4:**
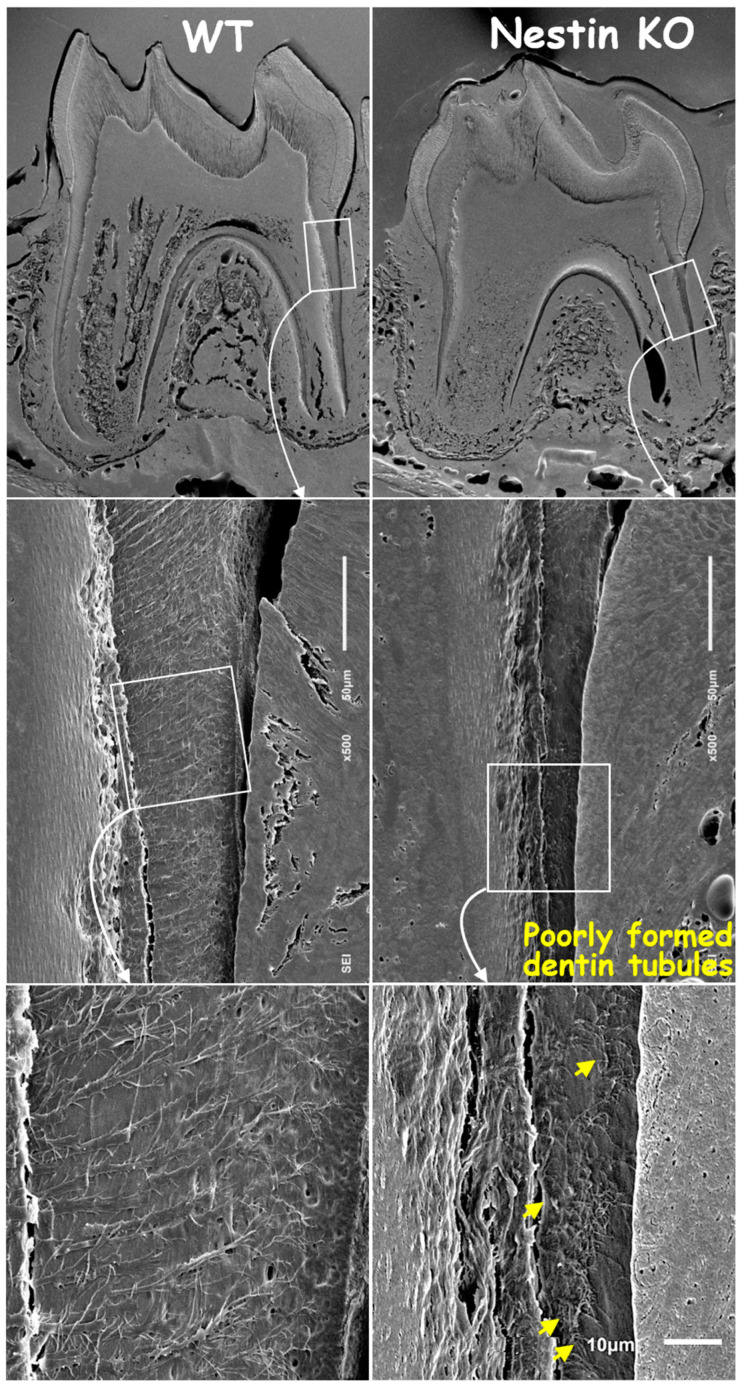
Acid-etched SEM images confirmed thinner root dentin, fewer and shorter dentin tubules, and poorly formed tubular structures in KO mice (yellow arrows).

Next, we removed mineral matrices using the acid-etched SEM technique and revealed poorly formed dentin tubules in KO mice, with a significant loss of tubular branches and disruption in original organization patterns compared to age-matched controls (Figure 4).

In addition, Backscattered (Supplementary Figure S1) and Acid-etched SEM (Supplementary Figure S2) images of the 2nd molar showed a great reduction in the root dentin thickness and tubules in Nestin KO mice, with no apparent change in crown dentin thickness. These findings are consistent with the results from the 1st molars. 

**Figure 5:**
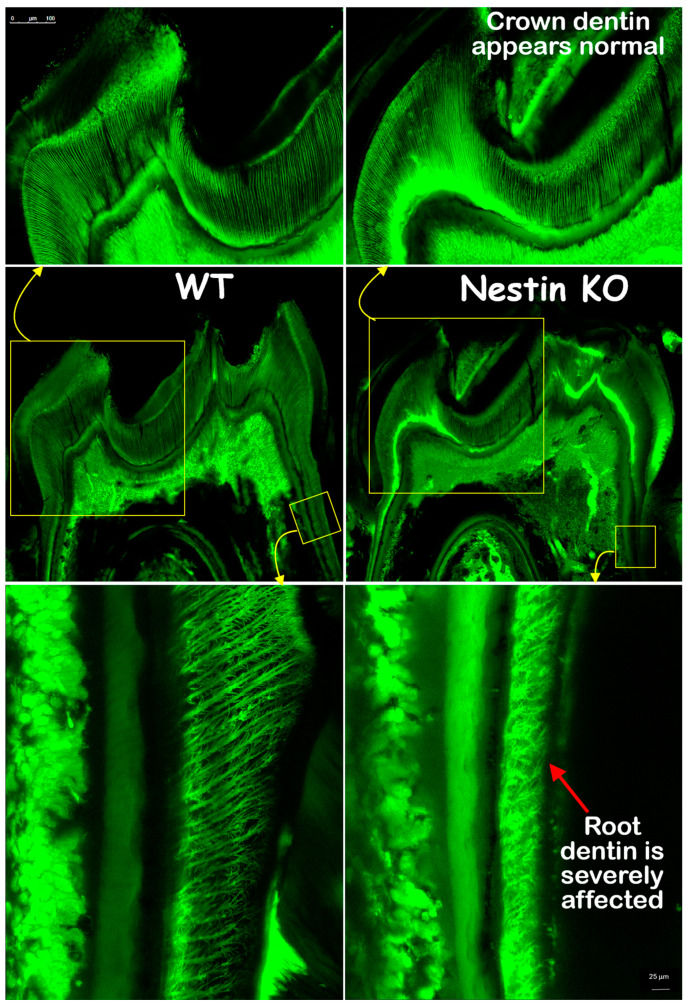
FITC images showed that crown dentin tubules (**upper panel**) in KO mice appeared similar to controls, while root dentin tubules (**lower panel**) were significantly affected, displaying reduced length and malformed branches.

The FITC images confirmed that the crown dentin formation of KO mice was not impacted, with dentin thickness and tubule distribution similar to those of the WT mice. However, the KO root dentin was severely affected, with much thinner dentin, shorter dentin tubules, and irregular tubular distribution and branches (Figure 5). In summary, these findings indicate that the root, but not the crown, dentin formation was severely impaired in KO mice.

### 3.3. Nestin KO Mice Developed Defects in Cellular but Not Acellular Cementum

**Figure 6:**
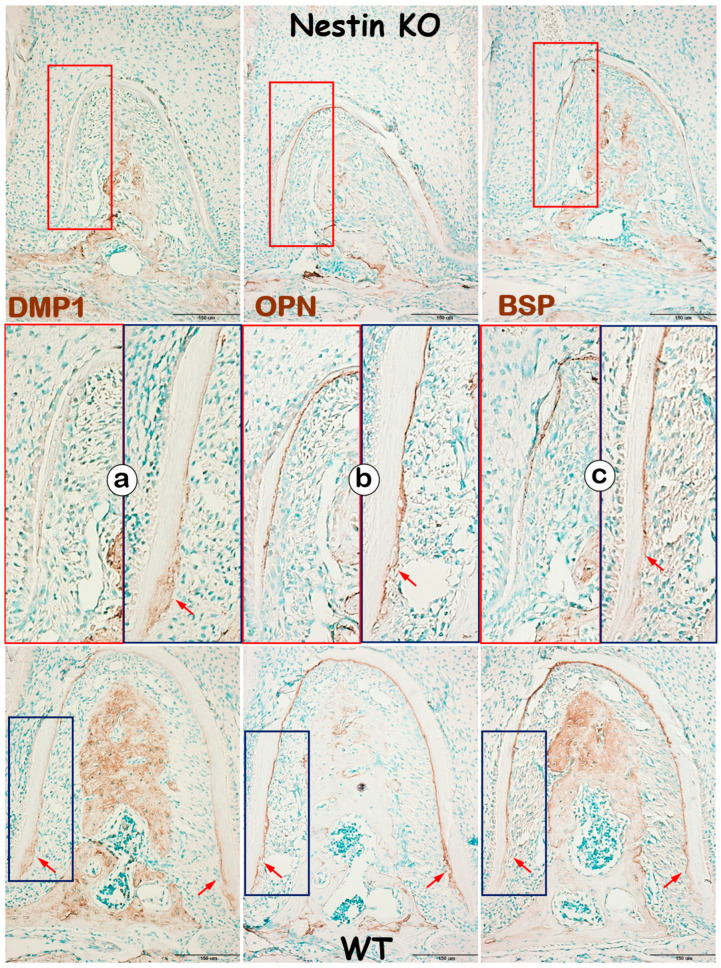
Nestin KO mice develop defects in cellular, but not acellular, cementum. (**a**) IHC images reveal a dramatic decrease in DMP1 expression in KO cellular cementum; (**b**) OPN expression remains similar between groups in the acellular cementum but is greatly reduced in the cellular cementum; (**c**) similarly, BSP is significantly affected in the cellular, but not acellular, cementum in KO mice. Red arrows indicating strong expression of DMP1, OPN and BSP in the cellular cementum of WT mice.

In addition to evaluating dentin, we examined changes in KO mice cementum, another mineralized tissue in roots, with IHC. DMP1, a matrix protein highly expressed in cellular cementum, was largely undetected in KO mice (Figure 6a). In contrast, OPN (Figure 6b) and BSP (Figure 6c), two matrix proteins expressed in both acellular and cellular cementum, showed no apparent change in KO acellular cementum but were greatly reduced in cellular cementum. These findings support the notion that Nestin plays an important role in regulating cellular cementum formation.

## 4. Discussion

The present study is the first to demonstrate that the deletion of Nestin results in dramatic changes in mouse molar root, but not crown, or dentin, and a decrease in cellular cementum mass, with little change in acellular cementum during postnatal development. These findings provide new insights into the molecular regulatory network underlying root formation, which has promising therapeutic value for tooth root regeneration.

Nestin plays a critical role in the prenatal and postnatal development of multiple tissues. It has also been reported to be involved in the interaction between epithelial and mesenchymal cells by facilitating the transition between these cell types during development, tissue repair, and cancer progression [21,22]. Particularly, the interaction of epithelial and mesenchymal cells is considered a critical mechanism for initiating root dentin formation [23]. This could partially explain the defects in dentin development observed in Nestin KO mice. Furthermore, our study also demonstrated that the lack of Nestin also impacts the formation of cellular cementum. A previous study reported that Nestin was expressed by progenitor cells in dental follicle tissue and is involved in cementoblasts differentiation [24]. Further studies are needed to clarify the role of Nestin in cementogenesis and to distinguish the developmental mechanisms underlying cellular and acellular cementum.

Since most of the root defects observed in the present study align with our previous findings in Osx conditional knockout (cKO) mice, we conducted a pilot study to investigate whether Nestin functions as a potential downstream molecule of Osx. We generated 2.3 Col 1-*Cre*; *Osx*^fx/fx^ mice by crossing *Osx*^fx/fx^ mice [25] with 2.3 Col 1-*Cre* mice [26] to facilitate the tissue-specific deletion of *Osx* in odontoblasts from the embryonic stage (Supplementary Figure S3a). The Nestin IHC results showed a great reduction in the expression of Nestin within Osx cKO root dentin (Supplementary Figure S3b), indicating that Nestin is likely a critical molecule in the *Nfic-Osx* pathway during tooth root formation.

The present study also found that osteoclastogenesis in the KO mandible was greatly impacted, as reflected by a marked reduction in TRAP^+^ osteoclasts and delayed tooth eruption. Previous studies have shown a strong association between Nestin and mesenchymal stem cells (MSCs), particularly Nestin^+^ bone marrow perivascular MSCs, which are closely linked to angiogenesis and bone resorption [12]. Further studies are needed to determine the role of Nestin in craniofacial bone formation and remodeling, as it may have significant implications for tooth development and diseases, dentition transition, and periodontal tissue health.

In summary, our study reveals a novel role of Nestin in the formation of tooth root dentin and cementum during postnatal development. An absence of Nestin causes malformation of the root, but not crown, or dentin, and a reduction in cellular cementum, indicating Nestin is likely a promising target specific for tooth root development and regeneration. Further studies are needed to elucidate the regulatory signaling pathways that control Nestin expression and function during postnatal tooth root development.

## Data Availability

All the data are presented in the manuscript and Supplementary File.

## Supplementary Materials

The following supporting information can be downloaded at: https://www.mdpi.com/article/10.3390/dj13030113/s1, Figure S1: Backscattered SEM images of the 2nd molar. Figure S2: Acid-etched SEM images of the 2nd molar. Figure S3: Nestin is likely a critical molecule in the *Nfic-Osx* pathway during tooth root formation.
