# Supplementary material for: Novel Roles of Nestin in Postnatal Root Formation
Source: Dent J (Basel). 2025 Mar 4;13(3):113. doi: 10.3390/dj13030113 (PMC11941000; doi:10.3390/dj13030113)
Supplement: Supplementary file 1 [file dentistry-13-00113-s001.zip › dentistry-3325116-supplementary.pdf]

Novel roles of Nestin in postnatal root formation

Yan Jing[1], Jinqiu Wu[1,2], Ying Liu[1], Xiaohua Liu[2,3], Chi Ma[4,5]

[1]Texas A&M College of Dentistry, Department of Orthodontics

[2]Texas A&M College of Dentistry, Department of Biomedical Sciences

[3]University of Missouri, Department of Chemical and Biomedical Engineering

[4] Scottish Rite for Children, Center of Excellence in Hip

[5]UT Southwestern Medical Center, Department of Orthopedic Surgery

Corresponding author

Yan Jing, Assistant professor

3302 Gaston ave, Dallas, Texas, USA, 75246

yjing@tamu.edu

Co-corresponding author

Chi Ma, Research scientist

2222 Welborn St, Dallas, TX 75219

chi.ma@tsrh.org

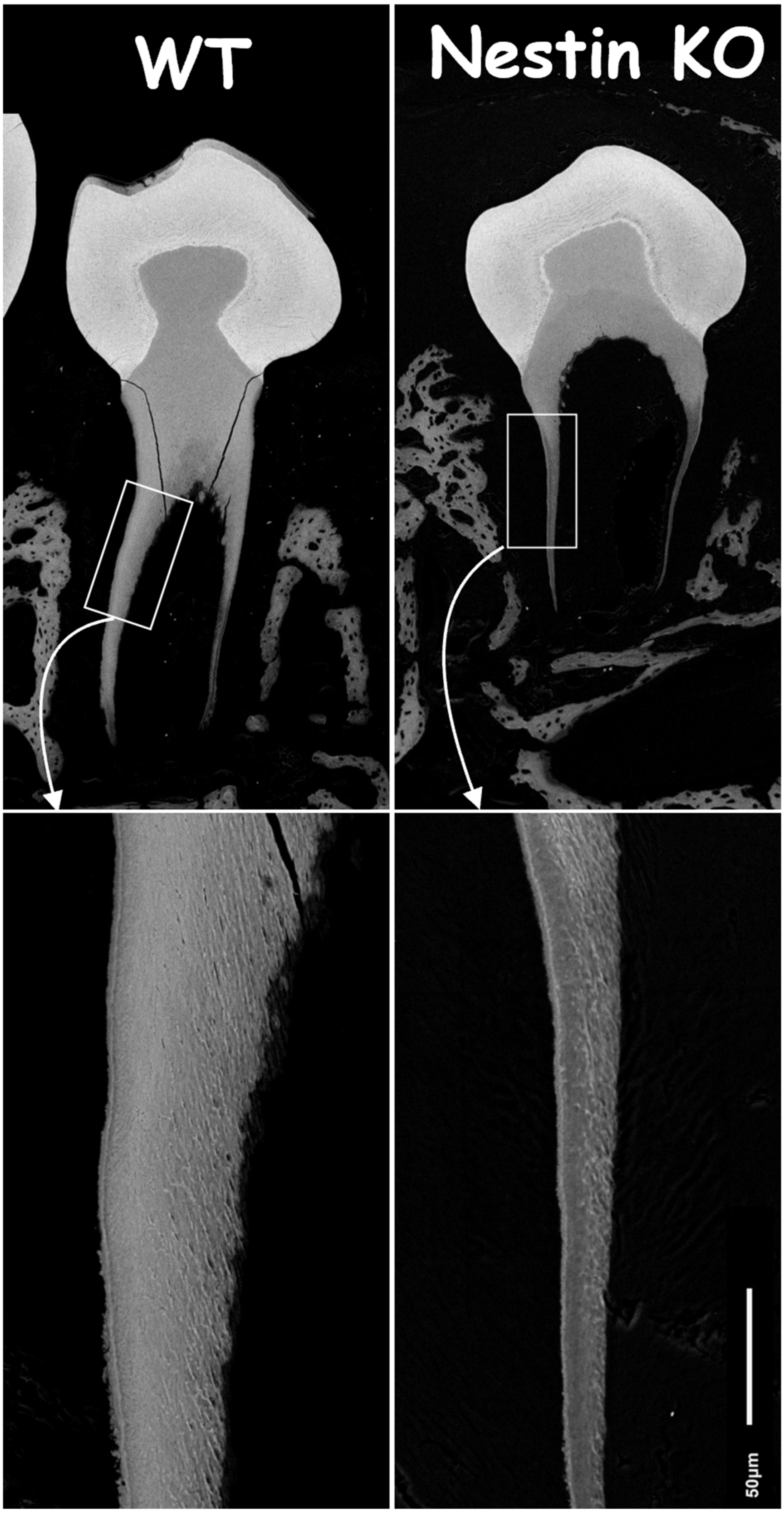

Figure S1. Backscattered SEM images of the 2nd molar showed a great reduction in the root dentin thickness in Nestin KO mice, with no apparent change in crown dentin thickness. These findings are consistent with the results from the 1st molars.

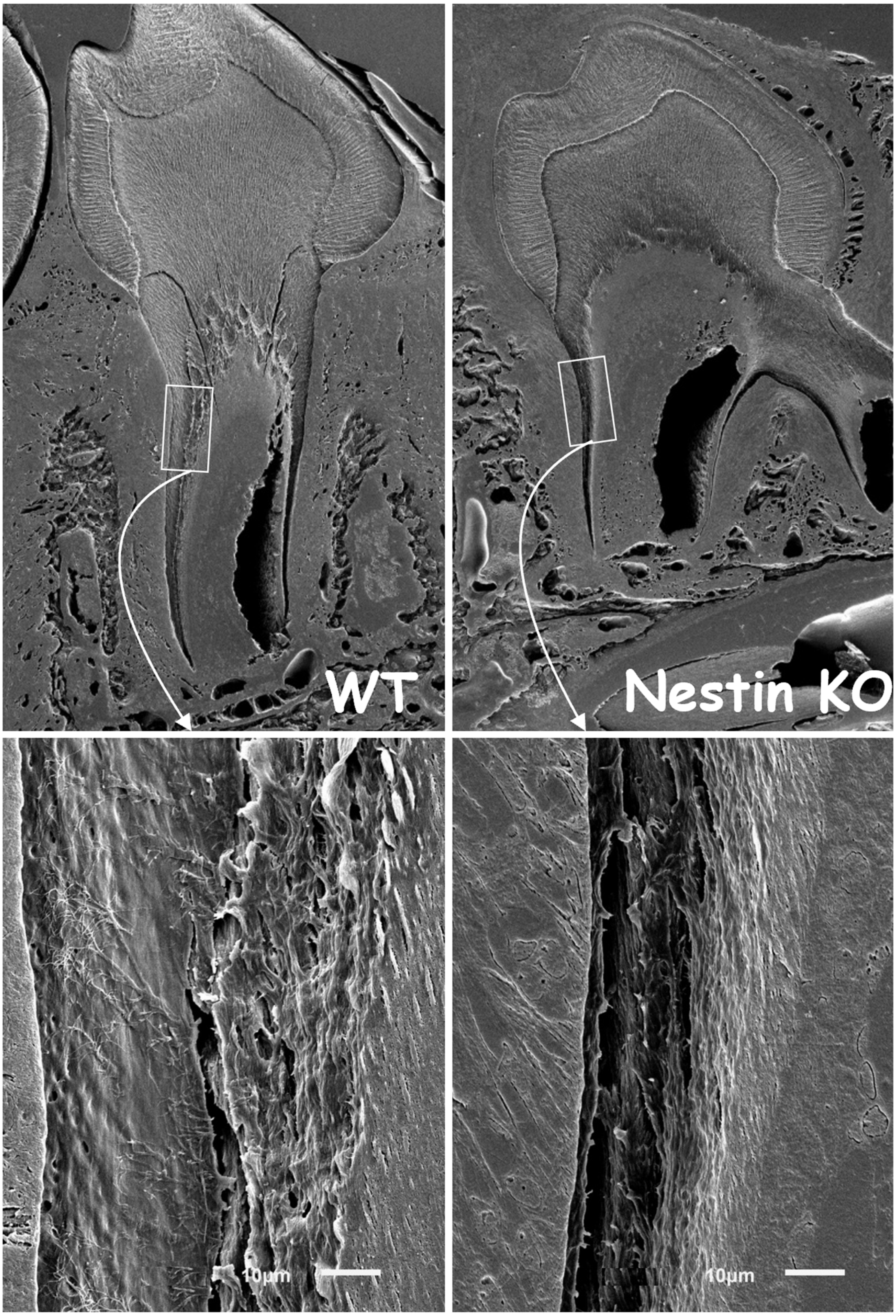

Figure S2. Acid-etched SEM images of the 2nd molar showed numerous dentin tubules in WT root dentin, which were absent in KO mice. Similarly, no apparent changes were observed in crown dentin structure.

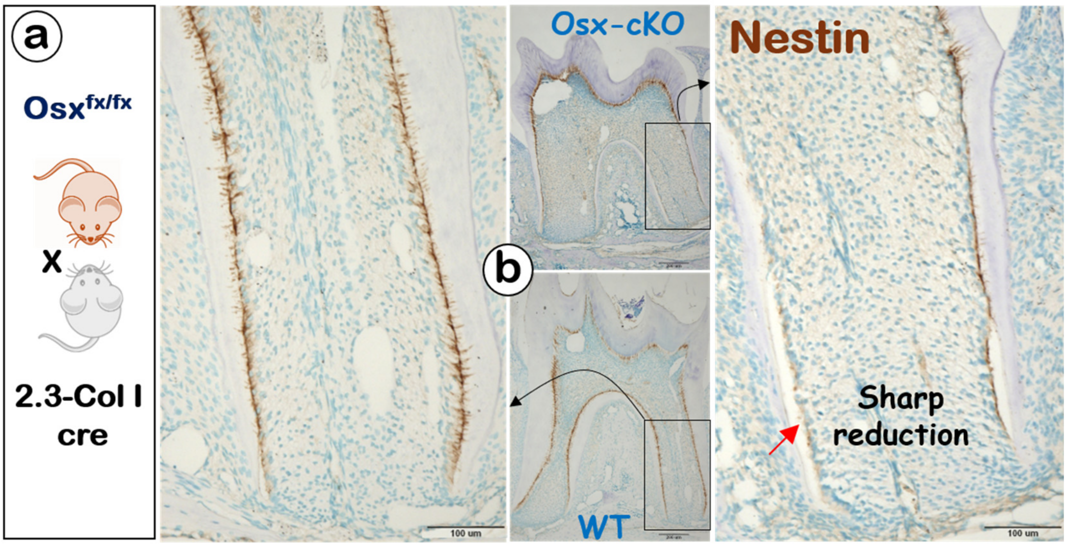

Figure S3. a) Osx cKO mice were generated by crossing 2.3 Col 1-*Cre* mice with *Osx*$^{fx/fx}$ mice and harvested at 4 weeks; b) Nestin IHC images showed a significant reduction in its expression in root odontoblasts of cKO mice compared to controls.
